# Interdisciplinary views of fNIRS: Current advancements, equity challenges, and an agenda for future needs of a diverse fNIRS research community

**DOI:** 10.3389/fnint.2023.1059679

**Published:** 2023-02-27

**Authors:** Emily J. Doherty, Cara A. Spencer, Jeremy Burnison, Marta Čeko, Jenna Chin, Lucca Eloy, Kerstin Haring, Pilyoung Kim, Daniel Pittman, Shannon Powers, Samuel L. Pugh, Demetris Roumis, Jaclyn A. Stephens, Tom Yeh, Leanne Hirshfield

**Affiliations:** ^1^Department of Computer Science, University of Colorado Boulder, Boulder, CO, United States; ^2^Institute of Cognitive Science, University of Colorado Boulder, Boulder, CO, United States; ^3^NIRx Medizintechnik GmbH, Berlin, Germany; ^4^College of Arts, Humanities, and Social Sciences, Psychology, University of Denver, Denver, CO, United States; ^5^Department of Computer Science, University of Denver, Denver, CO, United States; ^6^Department of Occupational Therapy, Colorado State University, Fort Collins, CO, United States

**Keywords:** fNIRS, diversity, bias, human-computer interaction, collaboration

## Abstract

Functional Near-Infrared Spectroscopy (fNIRS) is an innovative and promising neuroimaging modality for studying brain activity in real-world environments. While fNIRS has seen rapid advancements in hardware, software, and research applications since its emergence nearly 30 years ago, limitations still exist regarding all three areas, where existing practices contribute to greater bias within the neuroscience research community. We spotlight fNIRS through the lens of different end-application users, including the unique perspective of a fNIRS manufacturer, and report the challenges of using this technology across several research disciplines and populations. Through the review of different research domains where fNIRS is utilized, we identify and address the presence of bias, specifically due to the restraints of current fNIRS technology, limited diversity among sample populations, and the societal prejudice that infiltrates today's research. Finally, we provide resources for minimizing bias in neuroscience research and an application agenda for the future use of fNIRS that is equitable, diverse, and inclusive.

## 1. Introduction

Functional Near-Infrared Spectroscopy (fNIRS) is a portable, lightweight, and versatile non-invasive brain imaging modality that allows for the monitoring of brain activity by measuring changes in hemoglobin concentrations using optical light. It shares similarities with Functional Magnetic Resonance Imaging (fMRI), as they both measure hemodynamic activity in the brain. fNIRS has allowed neuroscience researchers to make strides by studying real-life scenarios, high workload environments, and face-to-face interactions (Hirsch et al., 2021), which are often unable to be studied using fMRI. Both fNIRS and fMRI measure changes in blood flow in the brain, however fNIRS has emerged as a popular approach as it is portable, captures both oxygenated and deoxygenated hemoglobin concentrations (Hoshi and Michael, 2005), has a higher tolerance to motion, and is much less expensive to operate than fMRI (Scarapicchia et al., 2017). As compared to Electroencephalogram (EEG), fNIRS, and EEG are complementary neuroimaging techniques; EEG measures changes in electrical changes in the brain while fNIRS measures changes in blood flow (Li et al., 2022). EEG lacks spatial resolution however presents higher temporal solution, whereas fNIRS has better spatial resolution and is restricted by its lower temporal solution caused by the slower hemodynamic response (Li et al., 2022). EEG is substantially lower in cost compared to fNIRS, however many manufacturers encourage and support the concurrent use of both technologies due to their complementary features (see Li et al., 2022 for a full comparison).

Since its development in the 1990s, fNIRS has progressed in terms of hardware and analysis techniques over the last three decades. Frans Jöbsis, an optical researcher, discovered the use of NIRS to monitor the hemodynamic activity of the brain during hyperventilation (Chance et al., 1962). The first studies using fNIRS investigated general cognition in relation to metabolism in the brain (Chance et al., 1992), differences in signal based on gender and handedness (Okada et al., 1993), and the effect of psychiatric disorders on brain activity (Okada et al., 1994). Originally, fNIRS devices recorded only single-channel data formed by one source and one detector, namely the first single-site instrument was the NIRO-1000 developed by Hamamatsu Photonics in Japan (Ferrari and Quaresima, 2012). Since then, fNIRS technology has advanced rapidly with newer, wearable, high-density systems developed all over the world from both large manufacturers and laboratory-developed systems including from Mexico (Gorostieta-Esperon and Jiménez-Ángeles, 2019), Japan (Kubo and Kubo, 2015), Europe (Piper et al., 2014; Pinti et al., 2015), China (Liang et al., 2016), and the U.S. (Ayaz et al., 2013; Tsow et al., 2021). Robust improvements in hardware have allowed investigation of unique domains not previously explored, such as movement-heavy activities like yoga (Dev et al., 2019; Dybvik and Steinert, 2021), unpredictable outdoor environments (McKendrick et al., 2016), and other naturalistic environments (Pinti et al., 2018), human-robot interaction (Le et al., 2022), collaborations between two or more agents (Czeszumski et al., 2020), and in sensitive populations (Arenth et al., 2007). Because these systems remain lightweight and portable, they are ideal for non-invasive brain measurement in a number of complex real-world scenarios (Le et al., 2022).

As the capabilities of fNIRS grow, however, so does the infiltration of bias in fNIRS research. Therefore, we gathered researchers spanning multiple disciplines including human-computer and -robot interaction, team science, software engineering, affect measurement in the brain and merging of fMRI/fNIRS modalities to study deep brain activity, as well as aging and injury research. Researchers were brought together to discuss their unique applications of fNIRS in their research, issues regarding fNIRS hardware and software, and how diversity, equity, and inclusion impact their research. This paper synthesizes the researchers' perspectives to glean commonalities and reviews each application domain represented to highlight its unique research questions, challenges, and expectations for the future. We also provide a unique perspective from a fNIRS manufacturer on the challenges and opportunities as they view the field in light of the diverse needs of their end user research populations.

While the researchers come from a diverse set of application domains, several common trends emerged: while analysis software and methods have drastically improved, there still lacks a standardized fNIRS methodology to be used universally, due in part to the frequent inaccessibility of educational resources. On the other hand, amelioration of hardware has allowed for investigation in novel scenarios and samples not previously accessible, and new high-density fNIRS systems now allow for comprehensive whole-brain measurement (Wang et al., 2021). Despite these improvements in fNIRS technology, a growing concern within the overall research community, especially in the field of neuroscience, is the presence of bias regarding the collection and diversity of data from limited populations (Webb et al., 2022). While technological advancements have allowed the exploration of new research spaces, the hardware itself makes it difficult to collect fNIRS data on certain populations including people with darker skin and hair (Kwasa et al., 2022) and those with sensory-motor issues (Arenth et al., 2007). Another avenue for bias is from prejudice and harmful views present in society that infiltrate this type of research in many ways (Roberts et al., 2020; Bradford et al., 2022), that will be explored later in the paper (see Section 4).

Another pressing and overarching concern repeatedly discussed by the researchers pertained to ethics in our scientific practices and the bias that is present in many fNIRS-based research pipelines (Yücel et al., 2021). It is expected that, because research samples are typically young, white, undergraduate students due to the lack of equitable research opportunities, bias is already present in our datasets (Roberts et al., 2020). We also examine the mechanisms behind societal and personal biases in neuroscience research and provide suggestions for identification and improvement.

The rest of this paper proceeds as follows: In Section 2, we describe how fNIRS is used in the interdisciplinary application domains of the researchers. In Section 3, we discuss the software and hardware challenges that researchers in these fields currently have with fNIRS and what implications these cause. We also discuss sources of bias in methodological pipelines of fNIRS research and how these can trickle down to adversely impact end users of fNIRS systems. In Section 4, we provide a perspective of these challenges and shortcomings in current fNIRS research, from the point of view of a well-established fNIRS manufacturer. Lastly, in Section 5, we summarize future needs of the community to address the challenges currently faced with respect to software, hardware, and bias in fNIRS research.

## 2. Sampling of current fNIRS application domains

In this section, we describe the myriad of application domains represented by the researchers, and how each domain currently uses fNIRS.

### 2.1. Collaborative and complex task environments

fNIRS is a promising sensor modality for research in collaborative and complex environments. Major advantages include its portability and flexibility as compared to other neuroimaging devices. For decades, the monitoring, maintenance, and enhancement of collaboration have been central research areas for cooperative environments and occupations. These environments and occupations have typically included, but are not limited to, industry organizations, the military, aerospace, education, hospitals, robot-operation, and natural disaster response, all of which can be complex socially, technically, and physically. Sensor and behavioral data are used to inform curriculum, design of technical specifications, team composition, and more with the overarching goal of maintaining or increasing healthy, productive, or efficient collaboration. fNIRS has shown merit in monitoring collaborative teamwork, including teams of humans and humans with robots.

#### 2.1.1. Collaborative problem solving

Collaborative problem solving (CPS) involves a group of two or more individuals engaging in a coordinated attempt to construct and maintain a joint solution to a problem (Roschelle and Teasley, 1995) and is considered a critical twenty-first century skill, as much of the complex work in the modern world is increasingly performed by teams (Graesser et al., 2018). Building on the rich theoretical advances in the science of CPS, recent research has turned its focus toward automatically analyzing CPS interactions with the help of machine learning (ML) and natural language processing. For example, researchers have used a variety of data streams [e.g., speech (Pugh et al., 2021), eye gaze (Abitino et al., 2022), body movements (Vrzakova et al., 2020)] collected during CPS to automatically identify the display of different skills (e.g., identifying when a team is constructing shared knowledge vs. resolving a disagreement through negotiation; Pugh et al., 2022). Such research often takes a multimodal approach (i.e., using data from multiple modalities), operating under the assumption that multiple data streams together (e.g., speech and body movement) can give a more complete picture of CPS processes than a single data stream (e.g., speech alone; Vrzakova et al., 2020).

With the advent of non-invasive brain imaging modalities, including fNIRS, to measure brain activity in realistic, face-to-face interactions, the field is beginning to explore the coordination of brain signals during social and collaborative interactions. Through studying these interactions, interbrain synchrony (IBS) has emerged as a behavior exhibited by teams during cooperative activities in which their brain signals become temporally coordinated (Hu et al., 2018). Synchrony reflects the attunement of one person to another's psychophysiological, cognitive, emotional, and behavioral state (Azhari et al., 2019). While we have learned some about IBS between two people, it has yet to be explored extensively in teamwork settings, and in relation to oneself. Literature is starting to emerge concerning how IBS may be able to predict collective performance in teams (Reinero et al., 2021) as detected by “hyperscanning,” the simultaneous recording neurological data of two or more people. Yet most of these works have been solely focused on educational settings (Bevilacqua et al., 2019; Davidesco et al., 2019; Davidesco, 2020). These studies have found increased learning flow and communication among students and teachers when their brain signals are in sync. Another scenario that has been studied concerns polarized political views: pairs of people with opposing views watched the same political video clip yet elicited different responses in their brains (van Baar et al., 2021).

Most hyperscanning studies cases have focused on assessing previously mentioned IBS, which has been implicated in joint attention, interpersonal communication, coordination, cooperation, and decision-making (Czeszumski et al., 2020). However, there is significant potential to move beyond IBS in this space. CPS involves a number of distinct processes that arise as a team works together to solve a problem (e.g., constructing shared knowledge vs. negotiating), and the neural mechanisms underlying these processes likely differ substantially. Future work could integrate the fNIRS hyperscanning paradigm with theoretically grounded annotations of CPS in order to investigate the differences in neural activity (both within a single brain and between teammates' brains) during these distinct processes. Additionally, incorporating fNIRS into CPS research is a promising step toward deepening our understanding of an understudied component of collaboration: the role of affect or emotion. However, available methodologies for objectively measuring these latent affective states, and investigating the ways that they contribute to (in)effective CPS, are limited. In future work, fNIRS could serve as a complement to existing methods (e.g., observational coding, self-report surveys) by providing a window into teammates' internal states, in order to investigate the role that affect plays in CPS.

#### 2.1.2. Mental state detection

The effect of complex tasks and environments on cognition can be better understood through the examination of one's mental state during said task or condition. fNIRS allows exploration of mental state during high-stress or high-intensity situations to better understand tasks that require high cognitive load. fNIRS has been used to study states including engagement and attention during flight simulation (Gateau et al., 2015, 2018; Liu et al., 2021), mental fatigue while driving (Ahn et al., 2016; Huve et al., 2018), the effect of mental stress on state classification (Al-Shargie et al., 2015; Al-Shargie, 2019; Katmah et al., 2021), and mind-wandering and visual attention (Durantin et al., 2015; Murata et al., 2015; Friedman et al., 2018). A better understanding of the various cognitive states during complex tasks can lead to in-the-loop systems that take fNIRS data as an input and act accordingly based on one's mental state. For example, one research group developed a neurofeedback system called NeuroDesignScience designed to take live fNIRS signals recorded during transmeridan flight on pilots to detect and mind-wandering and subsequently re-engage the pilot (Liu et al., 2021). As fNIRS continues to advance in terms of flexibility and accessibility, more feedback-loop systems could be designed for several occupations and scenarios.

#### 2.1.3. Human-robot interaction

Human-robot collaboration is becoming increasingly prevalent in many realms of complex and collaborative environments including rehabilitation, surgery, and the overall medical field (Raje et al., 2021), physical interactions in work (Smids et al., 2020), for those with cognitive (Scassellati et al., 2012) and physical (Van den Heuvel et al., 2016) disabilities and teamwork for task completion [Howell-Munson et al., 2022, for a full review of Human-Robot Interaction (HRI) applications, see Veling and McGinn, 2021]. Understanding how humans perceive a robot is salient in improving and increasing these interactions One unique application is to determine if and how people form a Theory of Mind of robots (Pittman et al., 2022). This research utilizes brain signals as an independent evaluation method of how humans perceive robots and compares the outcomes to computational tools like predictive ML that also seek to determine how people will perceive certain robots.

fNIRS provides the unique ability to probe the neural correlates of HRI as well as reveal the underlying mechanisms of psychological constructs essential to robot perception, such as Theory of Mind (Yorgancigil et al., 2022). There are very few mechanisms that allow machines to understand human behaviors, and fNIRS may provide a unique implicit (i.e., not self-reported) and real-time neurofeedback mechanism. This would provide novel insights toward the design and development of socially intelligent robots across multiple domains. fNIRS is a propitious technique to implement real-time human feedback that machines can understand to influence and ultimately improve HRI. The requirements for this address the current challenges around the application, feasibility, and reliability of fNIRS evaluations. For example, it can be experimentally tested how humans perceive distinct types of robots using fNIRS (Kawaguchi et al., 2012; Canning and Scheutz, 2013). From this, ML algorithms can be developed that can predict how a robot design is perceived, scaled to a larger amount of robots designs, and then be independently verified by comparing ML predictions with the fNIRS evaluation of the predictions as a baseline. This combination might allow for understanding human cognition in relation to robot perception and is crucial for implementing natural interaction capabilities in social robots. The potential impact of this research reaches from better understanding social robots and fNIRS capabilities to the implementation of better social robots in healthcare, elderly care, social assistance, and education (Hubbard et al., 2021; Kim et al., 2021; Pittman et al., 2022).

Robots show great promise in helping those varying socioeconomic status, in settings like schools and hospitals. However, many HRI studies study interactions with typically white and young samples, often sourced from universities, which are often not representative of the desired population (Leichtmann et al., 2022). A narrow sample may allow researchers to miss how cultural and racial differences impact HRIs. Lower socioeconomic status may be associated with more adversity to a robot due to lack of education and experience with such technology (Su et al., 2022). Many of these concerns surrounding lack of diverse sampling for HRIs also apply to the realm of collaborative problem solving, where current samples and in-laboratory tasks may not be representative of the rich and diverse collaborations that occur in real life.

### 2.2. Special populations and use cases

fNIRS research has been successful in measuring the brain activity of members of sensitive populations including those recovering from an injury, those with chronic illness who often experience negative affect, and other inherently disadvantaged populations. Exploring these subsets of participants and unique clinical cases with fNIRS has resulted in novel research findings and revealed limitations of using existing fNIRS technology to study historically underrepresented populations.

#### 2.2.1. Rehabilitation

Aging, injury, intervention, or a combination of these topics of research (e.g., following intervention in a population with brain injury) highly benefit from the investigation of cognitive workload associated with them. Studying the functional brain activity in those with physical disabilities or deficit can benefit these populations by designing tools to assist them or by better understanding the sequelae of their condition. For instance, there is great promise in rehabilitation tools that utilize a brain-computer interface for mobility assistance (Khan et al., 2018). An assistive system built to adapt to one's brain signal or mental state is yet another useful application of adaptive automation using neurotechnology, like fNIRS.

One specific use of fNIRS imaging in this area of research was to evaluate prospective changes in prefrontal cortex (PFC) activation following non-invasive brain stimulation and working memory training in healthy older adults. Older adults who completed working memory training and had improved working memory performance experienced reduced PFC activation during the working memory task, regardless of whether they received neurostimulation (Stephens and Berryhill, 2016). In this application of fNIRS, it was quite difficult, if not impossible, to acquire data from adults with thick or dark hair. Fortunately, because the sample was older adults, data acquisition was successful from most individuals because their hairlines had recessed over PFC. However, there were a number of participants from whom data could not be acquired, a significant limitation at the time. Furthermore, participants were required to remain seated during data acquisition, as a cart-based (i.e., non-mobile) fNIRS system was employed. In more recent applications of fNIRS, there has been increased success in acquiring data during movement-based tasks, which is essential for understanding neural underpinnings of functional movements. Specifically, fNIRS imaging has been paired with a dual task assessment, the Dual Task Screen (DTS), in athletes with and without sports-related concussion. The DTS requires athletes to complete an obstacle walk, a verbal fluency task, a hand-eye coordination task, and a mental math task (Aumen et al., 2020). Given the nature of these tasks, participants wear a mobile fNIRS device on their backs, which transmits data wirelessly to a laptop computer. Thus far, this device has supported acquisition of high-quality fNIRS data, and the data processing pipelines support identification and removal of motion artifacts (i.e., confounds). Still, data have been lost from some athletes with coarser hair and/or darker hair and skin, limiting the generalizability of findings.

#### 2.2.2. Clinical applications of fNIRS

fNIRS has also proven to provide several clinical applications including assisting the diagnosis and monitoring the treatment of Alzheimer's disease, schizophrenia, dyslexia, Parkinson's disease, childhood disorders, post-neurosurgery dysfunction, attention disorders, and more (Rahman et al., 2020). Whilst fNIRS has offered substantial findings in these applications, it is still not yet well-suited for clinical use. This is due to the device's flexibility, which allows for infiltration of the signal and cannot be trusted as completely accurate. fNIRS also only offers information about cerebral bloodflow, which could assist in the clinical process, yet it does not measure the rest of the brain including the deeper cortical structures (Irani et al., 2007). Evidence suggests that fNIRS may be used to explore brain functions and possibly assist clinicians with a faster and accurate diagnosis in the future (Rahman et al., 2020). fNIRS has not moved to real clinical practice yet mostly due to its lagged temporal resolution and susceptibility to motion and other physiological artifacts, which may lead to bias as we can only use fNIRS in lab-based environments for now (Chen et al., 2020). For a more detailed review of fNIRS clinical applications, please see Rahman et al. (2020).

#### 2.2.3. Affect measurement in the brain with fNIRS and fMRI

Affect is a fundamental property of brain function and is disturbed in people with chronic illness and pain (Hu and Gruber, 2008). In a recent study, Čeko et al. (2022) used multiple types of negative affect stimuli combined with fMRI and predictive modeling to identify brain patterns encoding common and stimulus type-specific negative affect that jointly shape our subjective experience. Arising from this foundational fMRI work is the need to incorporate ecologically valid approaches to better understand affective processes in naturalistic settings (i.e., “in-the-wild”) and for those with chronic illness who face unique challenges.

One such approach showing great promise is fNIRS. fMRI and fNIRS both explore brain activity by measuring blood flow in the brain. fNIRS can be used in naturalistic experimental contexts when fMRI is not available and affords slightly better temporal resolution. fNIRS being non-invasive is also beneficial to sensitive populations who often experience pain and negative affect who may not be able to modulate their real-life behavior in an fMRI scanner. However, commercial fNIRS devices have poor spatial resolution and cannot detect brain activity in deeper subcortical and inferior cortical regions. To enable neuroimaging researchers to take advantage of the complementary strengths of these two modalities, researchers aim to explore the correlation between these two types of signals and develop novel methods for predicting deep-brain activity as measured by fMRI from surface (cortical) activity measured by fNIRS (Liu et al., 2015). This will provide preliminary roadmaps for fNIRS researchers to leverage fMRI to improve modeling of brain data collected by fNIRS and for fMRI researchers to add ecologically valid experiments with fNIRS to their body of research. However, because of the inability of fNIRS to measure certain populations, there remains a gap in literature relating and comparing fMRI to fNIRS signals in these sensitive populations.

#### 2.2.4. Neurodevelopment and adolescent studies

The flexibility of fNIRS has allowed developmental researchers to explore many not well-understood cognitive processes in infants and young children. Several advances have been made in this research area with the use of fNIRS and we present some brief yet noteworthy findings. Most neuroimaging developmental studies, especially those using fMRI, are performed on sleeping or sedated infants (Wilcox and Biondi, 2015), however fNIRS is an alternative method that can be used on awake and engaged infants which has allowed for some crucial developmental findings. Among these notable studies the following have been studied in infants: facial processing (Ichikawa et al., 2019), language acquisition and development (Quaresima et al., 2012), visual and auditory attention (Emberson et al., 2017), and parent-infant relationships (Minagawa et al., 2018). Through the use of fNIRS, complex neurological phenomena in different contexts have been studied. For example, the effect of bilingualism on neurodevelopment have been studied on both infants (Blanco et al., 2021) and young children (Arredondo et al., 2017). Researchers have found differing prefrontal functional organization in English-Spanish bilingual children when compared to English-speaking children (Arredondo et al., 2017). For a full review of neurodevelopmental applications with fNIRS, please see Wilcox and Biondi (2015) and Azhari et al. (2020).

The use of fNIRS with infants and children still presents some challenges to researchers. Typically, the 10–20 international mapping system is used to place fNIRS probes corresponding to underlying cortical structures and this has been verified in adults (Tsuzuki and Dan, 2014), however it cannot be assumed that these cortical structures in children map as they do in adults (Wilcox and Biondi, 2015). Some researchers have coregistered cortical structures with fNIRS probes in infants, but only in specified areas including frontal, parietal, and temporal regions (Lloyd-Fox et al., 2014b). Still uncertain is the variability of hemodynamic responses among infants which poses challenges for researchers as well; some have found that the response is highly dependent on experimental design and stimulus type (Issard and Gervain, 2018).

#### 2.2.5. Understudied or marginalized populations

Certain populations including pregnant women, infants and adolescents, the elderly, and people with disabilities are often understudied due to limitations with traditional neuroimaging techniques like restricted movement and other health- or age-related restraints. These traditional neuroimaging approaches, such as fMRI and PET (positron emission tomography), also require participants to come to a neuroimaging site which increases the effort required to participate in a research study. The lack of mobility can prevent data collection from some participants with limited financial resources, unpredictable schedules, or inadequate caregiver support.

Compared to fMRI and PET, fNIRS offers mobility and safety that makes it an excellent approach for many of these understudied groups. First, fNIRS has been used to assess neural function in pregnant women (Roos et al., 2011). While many pregnant women express concern about multiple exposures to fMRI during pregnancy, they have minimal safety concerns about multiple assessments of neural function using fNIRS. Second, fNIRS has also been used with infants and young children. Compared to fMRI and EEG approaches, fNIRS is relatively less sensitive to the participants' motion. This helps the investigation of the brain activation in these young populations while they are awake and interact with robots or other individuals such as their parents (Lloyd-Fox et al., 2010; Wilcox and Biondi, 2015, see Section 2.2.4 for more). Third, the mobile fNIRS system minimizes participant burden by facilitating research at the participant's home or other convenient locations. For example, fNIRS has been used in examining the brain development of children in rural Africa (Blasi et al., 2019). The fNIRS system was brought to a rural village and the data was collected in a field station. While fNIRS improves the inclusion of the populations who were previously considered to be difficult to study and recruit, allowing these populations to be better represented in the neuroimaging literature, data collection in these less-constrained environments does not come without challenges. Researchers that have extended their work into understudied areas such as rural Africa (Lloyd-Fox et al., 2014a; Jasińska and Guei, 2018; Blasi et al., 2019) have reported technical issues including decreased signal quality due to sweating caused by extreme heat and the overheating fNIRS machines. Therefore, while the portability of fNIRS allows for collection in more convenient locations for richer, more diverse datasets, the data collection environment must be suitable for data collection.

Certain populations with disabilities may also benefit greatly from the merits of fNIRS technology, however there are often hardware limitations for these populations. One area of interest regarding fNIRS research is the study of auditory deficit and deafness, especially in those living with a cochlear implant (CI, Saliba et al., 2016). fNIRS has successfully been used in those with CIs yet not without unique challenges. Obtaining meaningful measurements of cortical activity has proven difficult in this population due to noise caused by CIs, therefore it is essential that during cap placement and design of optode layout, CI placement is considered (Saliba et al., 2016). Saliba et al. (2016) designed a custom fNIRS cap to not intrude with one's CI. This limitation is one that major fNIRS distributors should consider when designing caps.

Cognitive disabilities have also been studied with fNIRS, with much work focused on Autism Spectrum Disorder (ASD), a neurodevelopment disorder in which many of the neuromechanisms are yet to be understood (Zhang and Roeyers, 2019). One unique of fNIRS to this population include the ability to study neurodevelopment starting at a young age (Conti et al., 2022) to better understand the neural mechanisms of ASD. fNIRS is also advantageous compared to fMRI in this context as some with ASD can hardly control their hyperkinetic behavior or endure the enclosed space and loud noises (Zhang and Roeyers, 2019). Thus, fNIRS is a quieter and less-invasive method to use for those with these sensitivities. However, many of those with ASD struggle with sensory-motor issues (Piek and Dyck, 2004) that may prohibit them from wearing a fNIRS cap for an extended time or at all (Su et al., 2021). The development of remote, non-contact fNIRS systems where a cap is not required shows great promise for this population (Hirshfield and Meier, 2020).

Historically marginalized populations [e.g., the racially and ethnically underrepresented and low socioeconomic status (SES)] have been difficult to recruit for neuroimaging studies due to mistrust of participating in research, likely stemming from the historical harm and systemic inequities exclusive to marginalized populations (Dotson and Duarte, 2020). These groups tend to hesitate and even opt not to participate in studies using these approaches due to perceived risks (Scharff et al., 2010). There are also less research opportunities for these populations due to the fact that they are not representative of the general population (Roberts et al., 2020) causing these groups to be largely understudied.

While fNIRS offers solutions (i.e., portability, safety) to many of the obstacles posed by other techniques that prevent equal representation in research, the problems stemming in historical and societal biases are not as simple to solve. Going beyond the capabilities of fNIRS technology, these issues rooted in past discriminatory practices and a history of racism must be addressed on the systematic level, as echoed by Webb et al. (2022). Researchers, institutional review boards, scientific journals, and funding agencies have a shared responsibility to employ equitable research practices, including incentivizing the participation of marginalized groups and requiring demographic reporting (for more recommendations see Table 1 of Webb et al., 2022).

## 3. Discussion

By collecting input from the focus group of researchers spanning from many disciplines, we collated current challenges and concerns with fNIRS considering software, hardware, and the future of research.

### 3.1. Current limitations in fNIRS software

As echoed by researchers in varying fields, beginning research with fNIRS can be daunting due to inadequate open-source educational resources and lack of data pre-processing and analysis standards. As a community, we must improve openly available educational tools that are comprehensible for researchers not familiar with neuroscience imaging methods and that are inclusive of all populations. Because analysis method selection first begins with experimental design, education is essential to carrying out proper pre-processing and analysis to produce meaningful results. Several analysis software programs have emerged to pre-process and analyze fNIRS data, each having different capabilities, but most currently require at least mid-level expertise.

Regarding pre-processing, there is a multitude of filters and statistical methods available to pre-process fNIRS data, yet little documentation on how and when each method should be applied. Users must understand how the fNIRS signal should appear and when it is infiltrated with artifacts caused by motion or physiological confounds. These artifacts can create false results if not corrected during pre-processing. With many toolboxes and software options, users also find it hard to verify the steps made by the program. Modeling the signal is another step in which researchers must consider phenotypic characteristics of their sample pool. When modeling an fNIRS signal, there are several parameters that must be modified to properly convert the signal. Differential pathlength factor is one measure that must be modified based on age which converting the raw optical signal to hemoglobin concentrations using the modified Beer-Lambert Law (Kamran et al., 2018). Thus, it is important to modify pre-processing and analysis steps depending on the participant pool.

Both open-source and closed-source software options for analysis of fNIRS exist. Open-source resources, however, often do not offer ample documentation. Online communities and forums have helped new and existing users find support but have not solved this issue. Improvements have been made with processing software available in different programming languages, namely NIRS-SPM (Ye et al., 2009), Homer (Huppert et al., 2009), and NIRS AnalyzIR Toolbox in MATLAB (Santosa et al., 2018), and more recently MNE-NIRS in Python (Gramfort et al., 2014, for a full description of analysis software options, refer to Table 2 of Almajidy et al., 2019). These options are all viable, however new users have a difficult time choosing one over the other. Many researchers have questioned what advantages, if any, closed-source software options have over open-source resources. fNIRS analysis requires intermediate statistical knowledge, which is not widely practiced in many of the fields utilizing fNIRS in their research. Finally, although it appears that high-quality data can be acquired and there are good options for fNIRS data reduction and processing, there is still ambiguity surrounding optimal fNIRS data analysis.

Another gap in current analysis methods is the ability to analyze and interpret data in real time. In the years to come, there will be an increasing need to rapidly assess interactions with other agents [robots, artificial intelligence (AI), automated systems] and adapt the agent's behaviors appropriately. In order to achieve that, there needs to be real-time, machine interpretable human feedback that goes beyond objective measures. Further, real-time data analysis should be processed and displayed in user interfaces that aid direct individual or group decision making or fed into autonomous, virtual assistants that communicate with the human's suggested actions. To make the displayed data robust and reliable, software must have reliable and standardized methods for the pre-processing and analysis of fNIRS data.

Blood oxygenation likely will not be the only psychophysiological trend being monitored with wearable sensors during extreme conditions such as human spaceflight missions, so future extreme and dangerous habitats will need to sync concurrently collected physiological data, pair the information with environmental data, build models of workload and task performance, and effectively report findings. As we move into these more real-world and unpredictable environments, software must be developed to account for drift over long durations of sensor use or changes in physiological states of crewmembers. Thus, these systems must assess data quality in real time and either 1. pre-process and clean data or 2. make recommendations on how to adjust the sensors to improve data quality.

To educate the community, we encourage researchers to work on collating educational resources accessible to those new to fNIRS. As we learn more about the brain in different environments and scenarios, the need for a single repository for data storage and shared pipelines based on application use is evident. The creation of ERP CORE as a open-source resource and repository with example data pre-processing and analysis pipelines for analyzing EEG signals serves as an example of how an online fNIRS community could be built (Kappenman et al., 2021). A gold standard for fNIRS data processing should be created and shared within a user-friendly platform, like that of ERP CORE (Kappenman et al., 2021), to improve data acquisition and data sharing by researchers with and without computer science expertise. Recently, efforts have been made toward organizing fNIRS data into the standardized Brain Imaging Data Structure (BIDS) format (Gorgolewski et al., 2016).

### 3.2. Perspectives on fNIRS hardware

Whilst current fNIRS technology demonstrates great progress since the single-channel devices, newer hardware still has some limitations and poses challenges for certain populations. fNIRS technology uses optical light to measure hemodynamic activity in the brain *via* differences in optical absorption rates (Chen et al., 2020). Consequently, fNIRS devices struggle to represent data collected from individuals with dark, thick hair and/or darker pigmented skin, which only feeds the existing bias in the field of neuroscience (discussed in detail in Section 3.3). Ambient light interference (i.e., bright overhead lighting, sunlight) also makes it difficult to achieve optimal data quality “in the wild” (McKendrick et al., 2016).

Current gold standard fNIRS systems are costly, exist in form factors that are overly obtrusive when considering operational requirements in the field, and are not robust to motion (Tsow et al., 2021). While some commercial non-invasive wearable sensors exist that are better suited to the field than laboratory grade sensors, they are prohibitively costly considering normal “wear and tear” of extreme environments and are inadequate for use over long durations because of discomfort and variable reliability (Kyriakou et al., 2019). Portable fNIRS devices should provide enough light sources and detectors to allow for full-head coverage while maintaining a lightweight acquisition device that transmits data to a wirelessly connected computer. Many populations would benefit from hardware that is comfortable to wear for hours on end, simple to don, and robust to motion, sweat, skin oils, and other contaminants. This package would need to be integrated into other wardrobes such as extravehicular spacesuits, similar to how electrocardiogram functions can now fit into a smartwatch (Isakadze and Martin, 2020).

fNIRS holds promise for adaptive automation or to generate feedback that can be used in real time to calibrate human-agent interactions appropriately if the hardware technology advances to a point where fNIRS can generate reliable feedback as a wearable device. fNIRS systems should continue to support high-density measurements and for use in combination with fMRI and EEG, taking advantage of their unique properties. Current fNIRS systems are best suited for measuring brain activity from superficial cortical regions, which eliminates evaluation of deep-brain structures, which could be provided with concurrent fMRI measurement.

### 3.3. How bias plays a role in fNIRS research

Because of the conspicuous presence of bias existing on individual, institutional, and societal levels, it is vital to acknowledge the impact that bias has on research outcomes and how the exclusion of certain populations may exacerbate preexisting societal injustices. There are multiple sources of bias within neuroimaging and fNIRS research. The first source is implicit bias, a subtle form where individuals may be unaware of their discriminatory bias, often a result of being raised in a biased society, can manifest itself in the neural data of many (Stevens and Abernethy, 2018). For example, researchers found greater amygdala activity, a marker of fear, in response to people of color (Cunningham et al., 2004; Lieberman et al., 2005; Ronquillo et al., 2007). People of color are often associated with fear response because many are taught to fear based on someone's appearance, often stemming from early experiences (Stevens and Abernethy, 2018). The presence of implicit bias can result in unexpected brain activity, which raises the importance of reporting demographics like age, gender, race, etc. Researchers should accost the presence of implicit bias in their work by lessening the shame associated with personal ideals and acknowledging that personal bias may impact their findings.

Explicit bias, where one is aware of their prejudices, is often associated with feelings of shame, and those who are more explicitly biased are often better at controlling their reactions (Richeson and Shelton, 2003). Richeson and Shelton (2003) and Richeson and Trawalter (2005) found that regulation of racial bias taxes executive functioning resources, which are often studied in fNIRS research. This is further proof how personal bias can infiltrate the fNIRS signals when bias is not intentionally being explored. This again emphasizes why the presence of personal bias needs to be acknowledged as it may influence experimental results.

Recently, AI systems have been shown to produce biased and often derogatory results due to biased data being used to train ML algorithms within the human neuroscience domain (Parker and Ricard, 2022; Webb et al., 2022). Fairness in ML is a booming field, where researchers are looking to diversify their teams and their data to create more fair and equitable algorithms. A similar effort should be paid by the fNIRS community as not having equal representation of a certain population will result in other populations disproportionately or exclusively benefiting from the fruits of the research due to societal bias.

Because melanin affects light absorption, dark, thick hair, and darker pigmented skin affect fNIRS data collection. In fact, data collected on participants with darker skin and hair is often discarded due to bad data quality (Webb et al., 2022), creating a source of methodological bias. Popular devices such as smartwatches and pulse oximetry monitoring devices that use optical light face the same issue, leading to the exclusion of meaningful data from a significant percentage of the general population (Bradford et al., 2022). This has been an issue present in most research, as people of color are notoriously underrepresented in research, including general statistics datasets such as National IQ datasets (Sear, 2022) and clinical psychology (Bradford et al., 2022). fNIRS researchers must work to develop proper data collection techniques for underrepresented populations including ensuring proper cap size and using tools to optimize optode-to-skin contact.

It is essential to acknowledge the intersectionality of the many groups understudied with fNIRS. In 2021, the U.S. Census Bureau reported 19.5% of the country that live below the poverty line are Black (Creamer et al., 2022). In the same year, they reported that of Americans living with a disability, 25% live below the poverty line (Creamer et al., 2022). Several other disadvantages may lead to increased poverty rates such as employment status and level of education attained. Because of the challenges of participating in research and other factors, these populations continue be understudied and not supported by many neuroimaging technologies.

**Figure 1:**
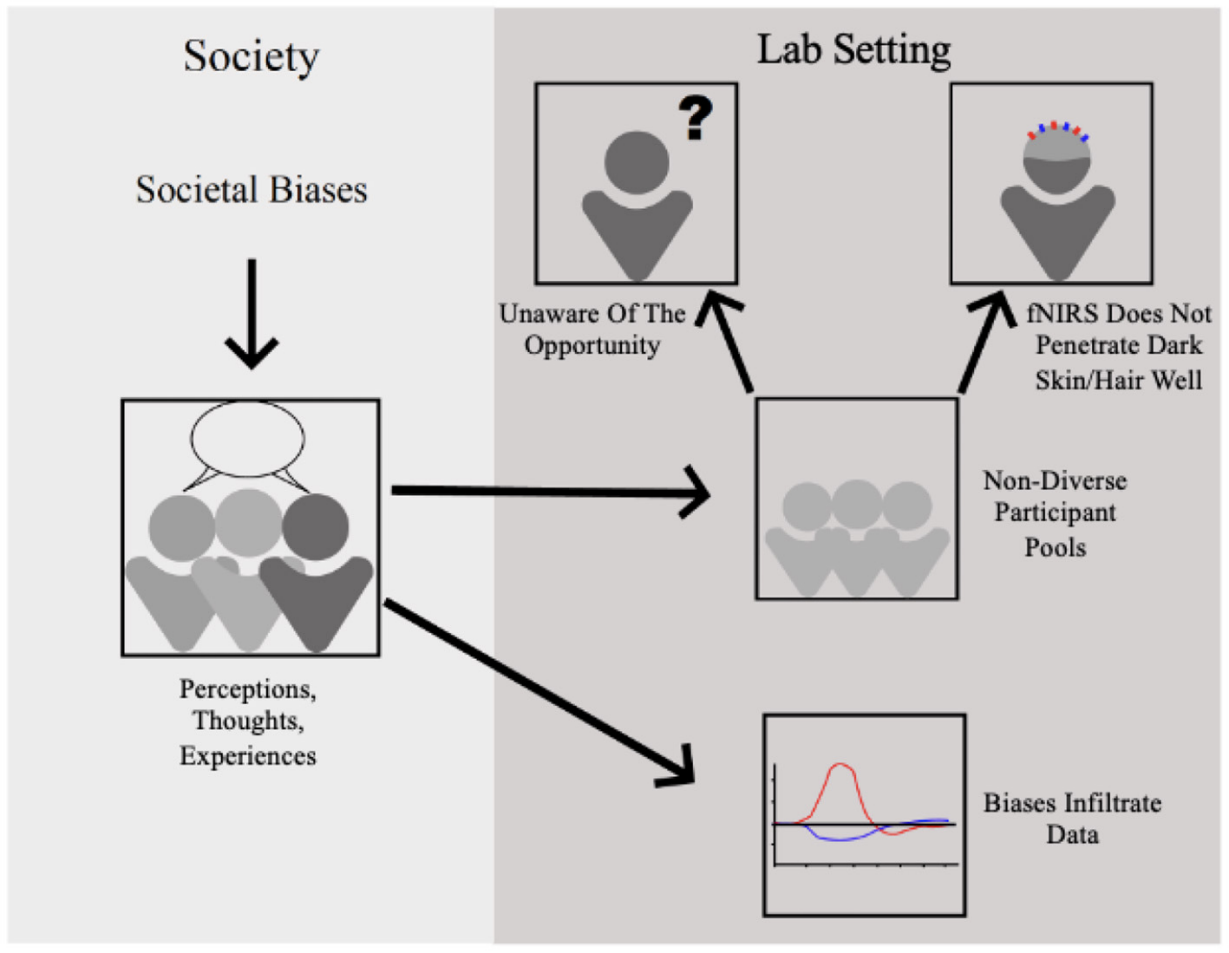
Biases present in society stemming from lived experiences and personal beliefs infiltrate the lab setting by effecting neurological data, providing small unrepresentative samples, and preventing important samples from providing data due to hardware limitations.

There are several ways that bias can infiltrate research (Figure 1). Societal biases, thoughts, opinions, and lived experiences all contribute to what is recorded in a lab setting by the means of infiltrating valuable neurophysiological data [i.e., heightened amygdala activity in response to people of color (Cunningham et al., 2004; Lieberman et al., 2005; Ronquillo et al., 2007) and maintaining of bias taxes executive functioning resources (Stevens and Abernethy, 2018)]. Another consideration is that participation in research studies is affected by socioeconomic disparities (i.e., not affiliated with an educational or research institution, insufficient compensation for missed work, limited research opportunities in certain areas of the world; Choy et al., 2022). Further, modeling interactions with limited populations leads to findings and technological advancements that are not applicable to all. For example, CPS is often observed and measured in groups that are similar in demographics (undergraduate students studying at the same university), and future work must investigate non-normative instances of CPS with more diverse groups that differ in demographics and lived experiences. Relatedly, positivity bias and significance bias, where failures or statistically insignificant results go unreported and unpublished for various socio-economic reasons, lead to interventions or measures seeming more effective or correct than warranted (Collaboration, 2015; Stanley et al., 2021). This can disproportionately affect populations those studies did not sample because the results and methods will become generalized in the literature despite being unreproducible with different samples (Roberts et al., 2020). To move beyond the racially, ethnically, and socioeconomically homogenous research samples, all researchers, including researchers in the fNIRS field, must address and aim to close this gap present in data (Dotson and Duarte, 2020).

We selected a subset of papers that explicitly accounted for phenotypic differences during analysis (Gemignani et al., 2018; Nagels-Coune et al., 2020) or directly investigated the effect of pigmented hair and skin on fNIRS signal quality (Fang et al., 2018; Bronkhorst et al., 2019). A closer examination of these selected papers (see Table 1) confirms that hair and skin color both significantly affect brain imaging results. This also demonstrates how few studies account for these effects in their analysis nor report skin and hair characteristics in their work that may be influencing results. While these papers offer practical advice to optimize the fNIRS signals in dark-skinned and -haired subjects, fNIRS researchers must consider effects due to phenotypic differences during data analysis, such as treating hair color as a fixed effect in a linear model (Gemignani et al., 2018).

**Table 1:** Brief literature review.

**References**	**Methods**	**Results**
Gemignani et al. (2018)	Performed a linear mixed effect model with hair as a fixed effect.	Model accuracy for deoxyHb using the generalized linear model GLM) was significantly higher for blonde subjects when compared to brown-haired subjects.
Nagels-Coune et al. (2020)	Studied how certain characteristics of hair (thickness, root density, and color) impact fNIRS results using a suitability questionnaire.	Subjects with lower suitability scores (based on hair/skin color) generally had more channels with poor signal-to-noise ratio.
Fang et al. (2018)	Used Monte Carlo simulation to study light propagation using a visible Chinese human corpse cryosection dataset.	Detected light intensity signal decreased by 15–80% when scalp hair follicle density varied from 1–11%.
Bronkhorst et al. (2019)	Studied how a simple head maneuver redistributed cerebral blood volume to verify if photon transmission is sufficient in darker-skinned subjects. They compared effects of a head tilt in a pigmented vs. non-pigmented subject.	Data from a pigmented and non-pigmented had comparable Hb patterns to effects of the head tilt. This tilt is recommended as a test to confirm photon transmission in subjects with pigmented skin.

## 4. Perspectives from creators of fNIRS solutions

As the fNIRS field is still maturing, creators of fNIRS solutions face plenty of exciting opportunities and motivating challenges. Taking an inclusive perspective, we presently express what we perceive as challenges and future directions shared by many manufacturers of research-grade fNIRS devices, with particular focus on the current researcher-motivated topics.

### 4.1. Phenotypic bias with hardware

Optimizing signal quality for participants in which typical caps and optodes do not work presents an important challenge to hardware development. Technical challenges often involve dark, thick, and curly hair styles that greatly increase the distance from optode to scalp and absorb a portion of the signal light (Bradford et al., 2022). This creates a gap in the literature studying those with cultural differences that keep them from wearing a typical fNIRS cap and optodes. Some inspiring ideas have been presented recently by the researcher community to help optimize the design of the culturally-sensitive optodes and caps to better accommodate these hairstyles, and we anticipate continued focus and advancements in the industry to mitigate this important problem. For example, in May of 2022, two neuroscientists published similar concerns on racial biases in the neuroscience domain (Parker and Ricard, 2022). In their article, they encourage the development of novel neuroimaging devices that overcome the issues that come with current technology such as the inability to penetrate through darker hair and skin. They advocate for the creation of fNIRS and EEG caps that are more accommodating to protective hairstyles (braids and twists) and coarse hair types by lengthening the probes and enlarging caps to cover thicker hair (Louis et al., 2022; Parker and Ricard, 2022). Technology must advance to accommodate cultural and phenotypic differences that are present all over the world. fNIRS creators must focus on such efforts as well as be transparent about how they are working to make fNIRS technology both more accommodating and accessible to diverse participant pools.

As the technology continues to develop, some of the current high-end fNIRS devices already contain features that help optimize signal quality in the context of the challenging phenotypes of darker hair and skin, including dynamic range adjustment for each individual optode and more sensitive light detection technology. However, there is still much to do to make fNIRS accessible to all hairstyles, textures, and colors, which is why multiple studies have recently launched with the aim of characterizing performance systematically in across participants with different hair and skin types to better understand the current state of the issue, identify opportunities for improvement, and direct further technology development (Nagels-Coune et al., 2020; Kwasa et al., 2022).

Another aspect of this issue involves the variability in the setup and troubleshooting strategy between end users. Educating researchers about the best practices for optimizing signal quality is a matter of maximizing the likelihood of success with various hair and skin types, and we will see a greater emphasis on training in this regard.

### 4.2. Analysis software standardization

While the NIRx Aurora fNIRS software (NIRx Medical Technologies) is one of the most widely used options for data acquisition, there has yet to be a similarly widespread convergence on fNIRS data analysis software for either real-time or offline processing that is beginner-friendly, professionally supported, and integrates the latest advanced approaches from signal processing through statistical analysis.

A recent addition to the data analysis software options, Satori by Brain Innovation (NIRx Medical Technologies), checks these boxes, but it is still challenged by its newcomer status and the divergence of analysis approaches that still characterize the fNIRS field. And whereas Satori is built for offline analysis, Turbo-Satori (Lührs and Goebel, 2017), another release from Brain Innovation, is designed for real-time applications such as brain-computer interfacing.

Although the foundational software exists, we need to enhance our community engagement and enrich the feedback stream that is crucial to optimize for application-specific solutions. Ultimately, building a robust end-user community and increasing efforts for analysis education and direct comparison of software toolboxes will facilitate standardization and best practices. And while the aforementioned toolboxes are commercial products, as has developed in many other scientific domains, the coexistence of open-source and closed-source software solutions for fNIRS will better serve the multitude of end-users and their varying requirements, expertise, and environments.

### 4.3. Future directions

Feedback from our extensive community of end users continues to drive us forward and navigate through development initiatives. With this compass, there is a lot to aim for in the future—portable, robust, lightweight, wireless, high-density fNIRS devices that can be used by all subjects and in all environments.

Currently, special detectors that contain internal gain amplification exist for special user-cases but are more expensive and bulkier than the more common non-amplifying type. In the future, ultra-sensitive detectors will be widely available in compact form, minimizing setup time and facilitating universal fNIRS access.

Furthermore, only a fraction of the current research groups goes through the process to derive the digitization of exact optode location on all their participants. In the future, every sensor location will be precisely known and registered to each individual's anatomy, reducing variability across recording sessions, subjects, and experiments. Digitization of optode locations on a per-subject basis, combined with sufficiently dense spatial sampling, would also make it more feasible to make small adjustments to individual optode locations to accommodate diverse hairstyles.

Reducing variability across sessions will greatly facilitate the expansion into new clinical and consumer markets, where recordings and results are less likely to be pooled across individuals. Beyond precise optode localization, it is likely that multiple fNIRS technologies, including continuous wave, time domain, and frequency domain, will be used to address this issue and clear the way for adoption in various settings. Expanding into new markets and increasing the scale of production will further reduce the cost of research devices and increase accessibility.

Lastly, the combination of fNIRS with another neural recording technology (e.g., EEG, fMRI) is greater than the sum of its parts. Simultaneously using multiple technologies allows for denoising of signals and cross-validation of results. Continued integration of modalities and standardization of associated analysis techniques are key challenges that will greatly benefit the universal fNIRS device of the future.

## 5. Conclusions and an agenda for future research

### 5.1. Future of fNIRS software and hardware

The community must also work toward establishing common pipelines for pre-processing and analysis based on varying experimental design. While there are several toolboxes and programs that carry out similar functions, there is little documentation on why certain functions or filters should be used depending on the data type and quality. Several of these tools make it difficult to truly understand what each function is performing on the data. This makes it difficult to recognize problems or abnormalities present in data. To standardize processing and analysis pipelines, there should be an open-source repository where data and pipelines can be stored, educational resources available to fNIRS beginners, and a community forum where researchers can collaborate. Efforts should also be made toward developing tools that better visualize hemodynamic activity in the brain (i.e., highlight what area of the brain is truly being activated).

Going forward, better standardization of data naming and organization, and processing pipelines are needed to be able to take the full advantage of this promising fusion of fNIRS and fMRI data. While the fMRI community has made substantial progress on both fronts, with the introduction of BIDS formatting for data naming and organization, and a push toward the use of a uniform pre-processing pipeline with excellent documentation and provenance (i.e., fMRIprep; Esteban et al., 2019), the fNIRS field seems to be lagging behind. We hope to see advances in this and a stronger culture of open sharing of fNIRS data, as is becoming standard (and often a requirement) for fMRI data, on platforms such as Open Neuro which currently hosts 550 fMRI datasets. Salient efforts to unite the community are underway with the formation of the Society of fNIRS (Yücel et al., 2017), and recent publications encouraging the standardization of fNIRS practices and the further unification of fNIRS researchers (Pinti et al., 2019; Yücel et al., 2021; Schroeder et al., 2022). These efforts may collectively raise awareness and develop tools concerning the issues of personal, societal, and methodological biases that impact fNIRS research.

While fNIRS hardware has changed rapidly over the last 30 years, there are still improvements to be made to harbor an inclusive and informative research community. Important next steps in the advancement of fNIRS hardware are to recognize the current gaps in data and subsequent literature and to find ways to optimize the signals for commonly marginalized populations. Working toward equitable fNIRS technology, where data collection is not restrictive of physical differences (such as skin and hair color), will minimize the methodological bias present in fNIRS research. The lack of inclusion of racial and ethnically diverse populations in literature must be acknowledged and addressed.

### 5.2. Application agenda for the future

Furtherance in software and hardware development is vital for the advancement of research within different application domains. Researchers across domains wish for hardware advancements that would allow for more “in the wild” feasibility to allow for investigation in high fidelity environments such as in the workplace (Martinez et al., 2022), outdoors (McKendrick et al., 2016), or even at home (Tsow et al., 2021). Understanding the brain mechanisms that drive everyday activities such as driving using a car-based fNIRS setup would help discover novel information about these processes like mind-wandering and distraction (Ogihara et al., 2022). An at-home consumer level fNIRS setup would broaden the applications of fNIRS research and make neuroscience research more accessible to all (Tsow et al., 2021).

To make fNIRS more inclusive, more attention should be paid to the underrepresented populations in which fNIRS is not a usable or efficient technology, including those with CIs and sensory-motor issues. We advise that either custom-made or customizable NIRS caps could help alleviate this issue. Also, the possibility of remote fNIRS should be further explored for those who cannot wear a cap (Hirshfield and Meier, 2020).

Researchers have also expressed the way for less expensive, more accessible ways to integrate fNIRS with compatible modalities such as fMRI and EEG. Both modalities are highly complementary to the fNIRS signal yet is not attainable to many due to lack of funding and lack of educational resources for going about research with multiple brain-monitoring technologies.

### 5.3. A call to address bias

As echoed throughout this paper, there is a dire need for researchers of all domains to address the bias present in their work. Specifically, to advance in the field of neuroscience, we must address how personal biases and lived experiences may impact neural findings, how societal and institutional level bias may prohibit the progression of research, how certain underprivileged populations are unaware of research opportunities or how research is purely inaccessible to many. Any researcher can make efforts to expand recruiting efforts to create more diverse participant samples, such as connecting with groups on campus and expressing the importance of their representation in science. We can look to the efforts made by the public health research and computer research communities to reduce the bias in their respective fields (e.g., Ford et al., 2018; Loi, 2021). Ethics committees and institutional review boards can require investigators to record the ways in which they are actively minimizing the bias in their work (Kwasa et al., 2022), such as Boston University and Boston Medical Center's Reducing Implicit and Explicit Bias in Research form (Boston University, 2022). Journals can reduce publication biases of prioritizing novelty, by accepting replication studies with more representative samples or modified methods and meriting participant diversity in the review process (Roberts et al., 2020). Moreover, modeling these efforts to junior researchers will help them become the norm so future science is more inclusive. Advocacy of hardware changes to accommodate sensitive populations is also required to make fNIRS data more inclusive.

In summary, through the exploration of several user-inspired research domains, we have revealed common limitations faced by researchers regarding fNIRS hardware and software. We also raise the issue of limitations caused by both implicit and explicit bias present throughout society and in lab settings. Lastly, we offer expert input on these issues and limitations from a group of fNIRS distributors and what they expect for future advancements in fNIRS technology and subsequent research. The future success of fNIRS research will rely on the resolution of issues raised in this paper as well as researchers working actively to reduce bias present in their research.

## Author contributions

ED led this paper, contributed to most of the writing, and made all revisions. CS helped with clarity, conciseness, and flow throughout the paper and contributed to writing on resources to address bias. LH advised and oversaw this project. The specific sections the authors contributed are: CS: Section 2.1. SPu and ED: Section 2.1.1. ED: Sections 2.1.2, 2.2, 2.2.2, and 2.2.4. DP, PK, KH, and JC: Section 2.1.3. JS: Section 2.2.1. MČ: Section 2.2.3. PK, SPo, TY, and ED: Section 2.2.5. DR and JB: Section 4. All authors reviewed and approved the final version.
